# Micromanaging checkpoint proteins

**DOI:** 10.7554/eLife.25001

**Published:** 2017-02-16

**Authors:** Andrea Ciliberto, Silke Hauf

**Affiliations:** 1 The FIRC Institute of Molecular Oncology (IFOM), Milan, Italy; 2 Department of Biological Sciences, Virginia Tech, Blacksburg, United Statessilke@vt.edu; 3 Biocomplexity Institute of Virginia Tech, Blacksburg, United States

**Keywords:** spindle checkpoint, kinetochore, protein kinase, cell cycle, *E. coli*, Human, *Xenopus*, *S. cerevisiae*, *S. pombe*

## Abstract

The kinase Mps1, long known to be the ‘boss’ in mitotic checkpoint signaling, phosphorylates multiple proteins in the checkpoint signaling cascade.

**Related research article** Ji Z, Gao H, Jia L, Li B, Yu H. 2017. A sequential multi-target Mps1 phosphorylation cascade promotes spindle checkpoint signaling. *eLife* **6**:e22513. doi: 10.7554/eLife.22513

Micromanagement has a bad reputation. It is frowned upon in the workplace, but it may have benefits in cellular signaling because a single regulator that interferes at multiple steps of a signaling cascade can lead to more reliable signaling. Now, in eLife, Hongtao Yu and colleagues at the University of Texas Southwestern Medical Center – including Zhejian Ji and Haishan Gao as joint first authors – report that a kinase called Mps1 acts as a micromanaging boss of a checkpoint signaling pathway that regulates cell division (Ji et al., 2017).

When cells divide, their chromosomes duplicate and a protein complex called the kinetochore assembles on each chromosome copy. Microtubules then attach to the kinetochores to pull the copies apart and segregate them between the newly forming cells. The mitotic checkpoint is a cellular safeguard that triggers the checkpoint signaling cascade if the microtubules do not attach properly to the kinetochores. In particular, this cascade leads to the formation of the “mitotic checkpoint complex”, which inhibits another multi-protein structure called the anaphase-promoting complex. This inhibition prevents chromosome segregation and the final stages of cell division (Musacchio, 2015).

Hints that Mps1 oversees and controls checkpoint signaling were uncovered decades ago. The overexpression of Mps1 was found to trigger checkpoint signaling even when the microtubules were all properly attached to kinetochores (Hardwick et al., 1996). In order to create the checkpoint signal, Mps1 relied on all other known checkpoint proteins, which suggested that Mps1 is the boss at the top of the signaling cascade.

Over the years, it became clear that Mps1 phosphorylates multiple checkpoint proteins, and also the kinetochore protein KNL1, but the mechanistic details of these events have only recently started to emerge. Phosphorylation of KNL1 leads to the recruitment of the checkpoint protein Bub1 to kinetochores. And work in budding yeast subsequently revealed that Mps1 phosphorylates Bub1 to enable it to bind to another checkpoint protein called Mad1. This interaction was crucial for checkpoint signaling in budding yeast (London and Biggins, 2014), but efforts to detect such an interaction in other organisms were unsuccessful. Now, however, Ji et al. provide strong evidence that a similar interaction occurs in human cells. Similar findings have emerged from research into fission yeast (Mora-Santos et al., 2016; Yuan et al., 2017).

The work of Ji et al. goes further by showing that Mps1 also phosphorylates Mad1 (as opposed to just phosphorylating Bub1 so that it can bind to Mad1). The region of Mad1 that is phosphorylated was known to have an essential role in checkpoint signaling, but its precise function had remained unclear (Heinrich et al., 2014; Kruse et al., 2014). Ji et al. now find that this region binds a protein called Cdc20 that has a central role in cell division as the activator of the anaphase-promoting complex. Checkpoint signaling packs Cdc20 into the mitotic checkpoint complex, thereby blocking its activity: however, this can only happen if Cdc20 first binds to a spindle checkpoint protein called Mad2. This binding occurs in an unusual fashion, with Mad2 changing conformation as it closes around a flexible fragment of Cdc20, just like a car seatbelt wrapping around a passenger. Ji et al. now propose that Mps1-phosphorylated Mad1 positions the flexible Cdc20 segment for capture by Mad2 (Figure 1). This is an intriguing model, and it will be important to corroborate it by structural or biophysical methods.

**Figure 1.**
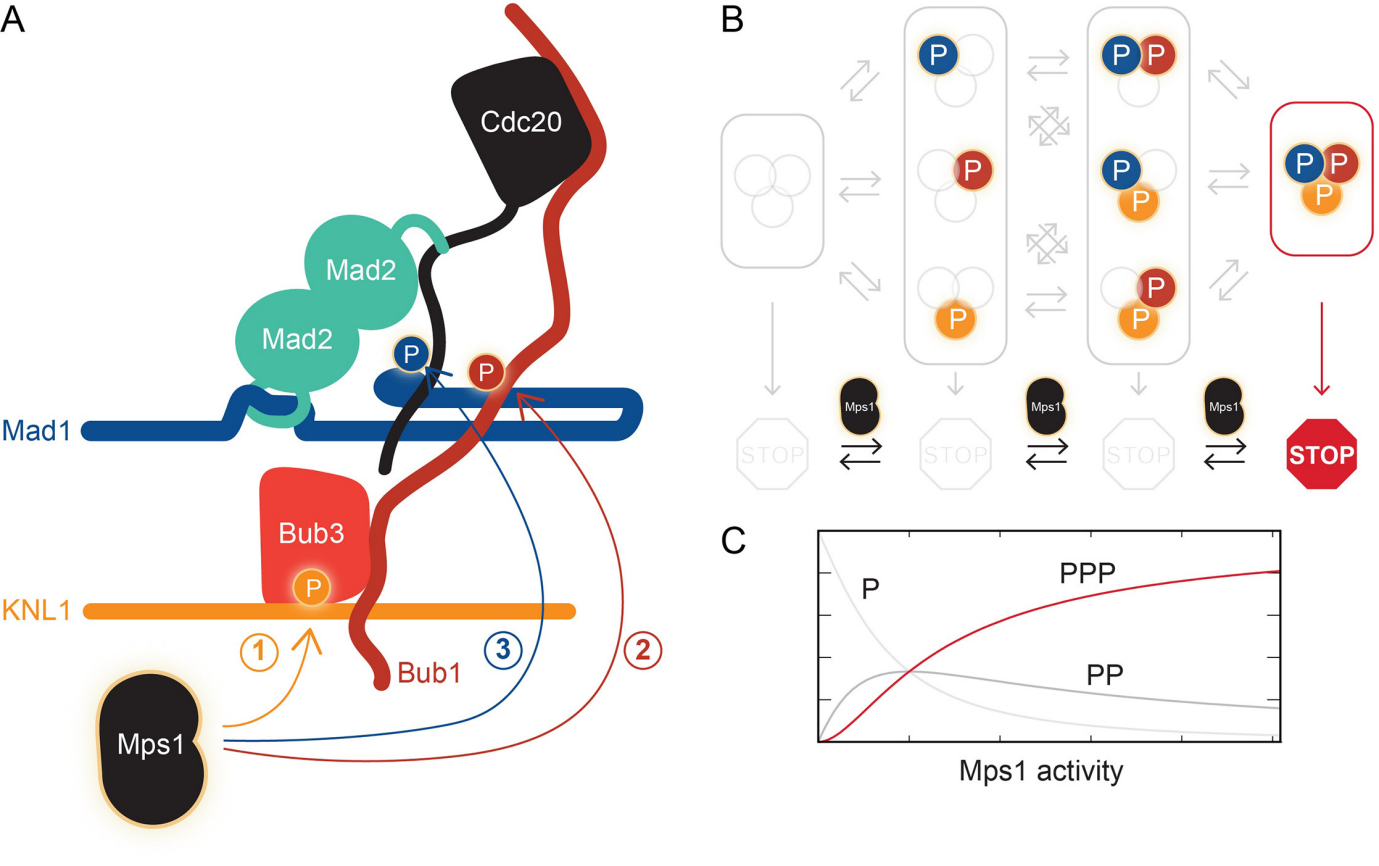
The kinase Mps1 and its role in mitotic checkpoint signaling. (**A**) Mps1 phosphorylates (P) three different proteins to promote the assembly of the mitotic checkpoint complex. It phosphorylates the kinetochore protein KNL1 to recruit the checkpoint protein complex Bub1-Bub3 to KNL1 (1). It phosphorylates Bub1, which allows this protein to interact with another checkpoint protein, Mad1 (2). It also phosphorylates Mad1, which promotes the binding of Mad2 to the regulatory protein Cdc20 (3). Ji et al. propose that phosphorylated Mad1 binds to Cdc20, thereby positioning the latter for capture by Mad2. (**B**) The checkpoint (represented by the STOP sign) is only active when Mps1 has phosphorylated all three proteins, KNL1, Bub1, and Mad1. (**C**) Checkpoint activity (y-axis) plotted as a function of Mps1 kinase activity (x-axis) for the phosphorylation of one (P), two (PP) or all three sites (PPP).

Further support for this model comes from a recent study that used a technique called FRET (which probes the distance between fluorescently labeled molecules) to follow the assembly of the mitotic checkpoint complex over time (Faesen et al., 2017). This work demonstrated that the binding of Mad2 to Cdc20 is the rate-limiting step in the assembly process, and that the phosphorylation of Mad1 by Mps1 is crucial for the process to occur efficiently. The data from both reports contain many other gems for mitotic checkpoint aficionados, and we encourage all checkpoint enthusiasts to take a read.

But back to micromanagement. Is it significant that Mps1 influences multiple interactions throughout the checkpoint signaling pathway? The phosphorylation of multiple substrates in a single pathway conceptually resembles the phosphorylation of a single substrate at multiple sites. As opposed to a single phosphorylation event, multi-site phosphorylation can lead to more interesting behaviors (Ferrell and Ha, 2014). In particular, the output of the signaling pathway can be negligible when the activity of the Mps1 kinase is low, but it can increase abruptly if kinase activity rises above a certain threshold (Figure 1C). This could prevent spurious Mps1 kinase activity from activating the checkpoint at the wrong time. Ji et al. speculate that Mps1 phosphorylates one substrate after the other in a “cascade”, though this remains to be tested.

Lastly, if you have a micromanaging boss, you want to keep her or him in check. Close contact between Mps1 and the kinetochore protein KNL1 is sufficient to trigger checkpoint signaling, even if there are no unattached kinetochores in the cell (Aravamudhan et al., 2015; Yuan et al., 2017). Hence, for the checkpoint to work properly, Mps1’s access to KNL1 needs to be under tight control and should only happen if there is a problem with microtubule attachment to kinetochores. Despite some initial work, this crucial part of the regulation is still only partly understood (Aravamudhan et al., 2015; Hiruma et al., 2015; Ji et al., 2015).

## References

[bib1] Aravamudhan P, Goldfarb AA, Joglekar AP (2015). The kinetochore encodes a mechanical switch to disrupt spindle assembly checkpoint signalling. Nature Cell Biology.

[bib2] Faesen AC, Thanasoula M, Maffini S, Breit C, Müller F, van Gerwen S, Bange T, Musacchio A (2017). Basis of catalytic assembly of the mitotic checkpoint complex. Nature.

[bib3] Ferrell JE, Ha SH (2014). Ultrasensitivity part II: multisite phosphorylation, stoichiometric inhibitors, and positive feedback. Trends in Biochemical Sciences.

[bib4] Hardwick KG, Weiss E, Luca FC, Winey M, Murray AW (1996). Activation of the budding yeast spindle assembly checkpoint without mitotic spindle disruption. Science.

[bib5] Heinrich S, Sewart K, Windecker H, Langegger M, Schmidt N, Hustedt N, Hauf S (2014). Mad1 contribution to spindle assembly checkpoint signalling goes beyond presenting Mad2 at kinetochores. EMBO Reports.

[bib6] Hiruma Y, Sacristan C, Pachis ST, Adamopoulos A, Kuijt T, Ubbink M, von Castelmur E, Perrakis A, Kops GJ (2015). CELL DIVISION CYCLE. Competition between MPS1 and microtubules at kinetochores regulates spindle checkpoint signaling. Science.

[bib7] Ji Z, Gao H, Jia L, Li B, Yu H (2017). A sequential multi-target Mps1 phosphorylation cascade promotes spindle checkpoint signaling. eLife.

[bib8] Ji Z, Gao H, Yu H (2015). CELL DIVISION CYCLE. Kinetochore attachment sensed by competitive Mps1 and microtubule binding to Ndc80C. Science.

[bib9] Kruse T, Larsen MS, Sedgwick GG, Sigurdsson JO, Streicher W, Olsen JV, Nilsson J (2014). A direct role of Mad1 in the spindle assembly checkpoint beyond Mad2 kinetochore recruitment. EMBO Reports.

[bib10] London N, Biggins S (2014). Mad1 kinetochore recruitment by Mps1-mediated phosphorylation of Bub1 signals the spindle checkpoint. Genes & Development.

[bib11] Mora-Santos MD, Hervas-Aguilar A, Sewart K, Lancaster TC, Meadows JC, Millar JB (2016). Bub3-Bub1 binding to Spc7/KNL1 toggles the spindle checkpoint switch by licensing the interaction of Bub1 with Mad1-Mad2. Current Biology.

[bib12] Musacchio A (2015). The molecular biology of spindle assembly checkpoint signaling dynamics. Current Biology.

[bib13] Yuan I, Leontiou I, Amin P, May KM, Soper Ní Chafraidh S, Zlámalová E, Hardwick KG (2017). Generation of a spindle checkpoint arrest from synthetic signaling assemblies. Current Biology.

